# Genetic polymorphisms in leptin, adiponectin and their receptors affect risk and aggressiveness of prostate cancer: evidence from a meta-analysis and pooled-review

**DOI:** 10.18632/oncotarget.12747

**Published:** 2016-10-19

**Authors:** Meng-Bo Hu, Hua Xu, Ji-Meng Hu, Wen-Hui Zhu, Tian Yang, Hao-Wen Jiang, Qiang Ding

**Affiliations:** ^1^ Department of Urology, Huashan Hospital, Fudan University, Shanghai 200040, China

**Keywords:** leptin, adiponectin, prostate cancer, genetic polymorphism, meta-analysis

## Abstract

Leptin and adiponectin signaling was associated with development and progression of various cancers. The present study aimed to clarify the role of genetic variants in leptin, adiponectin and their receptors in prostate cancer. After comprehensive search and manuscript scanning, a total of 49 genetic variants were enrolled and examined for their relations to cancer risk and aggressiveness. In the meta-analysis, *LEP* rs7799039 (allele contrast: OR 1.133, 95%CI 1.024-1.254), *ADIPOQ* rs2241766 (allele contrast: OR 1.201, 95%CI 1.015-1.422) and *ADIPOR1* rs10920531 (allele contrast: OR 1.184, 95%CI 1.075-1.305) variants were identified to be correlated with increased risk of prostate cancer. On the contrary, *LEPR* rs1137101 (allele contrast: OR 0.843, 95%CI 0.730-0.973) and *ADIPOR1* rs2232853 (allele contrast: OR 0.638, 95%CI 0.535-0.760) variants were associated with decreased risk of prostate cancer. From the pooled-review, we additionally recognized eight variants associated with cancer risk and another eight variants associated with cancer aggressiveness, respectively. These observations indicated important roles of leptin, adiponectin and their receptors in the development and progression of prostate cancer. The identified polymorphisms might assist in developing better risk-assessment tools, as well as generating novel targeted therapies, especially for obese cancer patients with impaired leptin and adiponectin signaling.

## INTRODUCTION

Prostate cancer (PCa) recently became the second most frequently diagnosed malignant tumor, and the fifth leading cause of cancer death in men throughout the world [1]. Extensive evidence showed that obesity was implicated in the onset and progression of PCa [2, 3], and increasing researches were conducted to discover the connections between them. Of note, the aberrant secretion and signaling of various adipokines, including leptin and adiponectin, was proved to be one of the plausible mechanisms for carcinogenesis in obese man [4, 5].

Epidemiological evidence showed that high levels of serum leptin (*LEP*) were associated with onset and progression of various cancers [4, 6, 7]. *In vitro* studies revealed that leptin and its downstream signaling might induce cell proliferation [8], inhibit apoptosis [9], and mediate metabolism [10]. On the contrary, study showed that adiponectin (*ADIPOQ*) inhibited proliferation and invasion of PCa cells [11]. Moreover, the adiponectin level was proved lower in patients with metastatic PCa compared to those with organ-confined diseases [12]. Both leptin and adiponectin relied on binding to their receptors, leptin receptor (*LEPR*) or adiponectin receptor 1/2 (*ADIPOR1/ADIPOR2*), to activate downstream signaling and functioning [13, 14].

By modulating the levels and functioning of leptin and adiponectin, the genetic factors (single nucleotide polymorphisms and microsatellites in *LEP*, *LEPR*, *ADIPOQ*, *ADIPOR1* and *ADIPOR2*) were demonstrated to affect the risk and aggressiveness of PCa [15–17]. Nevertheless, these studies were still insufficient and inconsistent. Therefore, we conducted an updated meta-analysis and pooled-review to better clarify the role of genetic variants in leptin, adiponectin and their receptors in PCa. The present study aimed to help developing better risk-assessment tools and targeted therapies in the field of PCa.

## RESULTS

### Study characteristics in the meta-analysis and pooled-review

**Figure 1:**
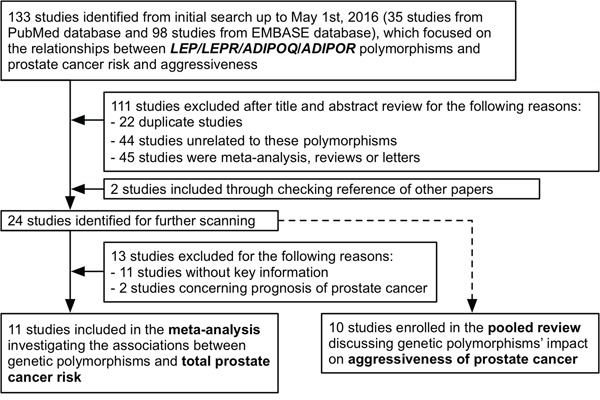
Flow chart of study selection and identification

As shown in Figure 1, a total of 133 published studies were identified from different database after initial search. We excluded 111 studies after title and abstract scanning, and enrolled two additional studies through manual reference searching. Then, a total of 24 studies were subject to full-text examination. Consequently, a total of 11 studies [18–28] were enrolled in the meta-analysis investigating the relationships between *LEP*/*LEPR*/*ADIPOQ*/*ADIPOR1*/*ADIPOR2* variants and total PCa risk (Table 1), and ten studies [19, 23, 29–36] were included in the pooled-review discussing these polymorphisms' impact on the aggressiveness of cancer (Table 2).

**Table 1:** Characteristics of studies included in the meta-analysis

Author	Year	Country	Ethnicity	Control source	Genotyping methods	Size (case/control)	Genetic polymorphisms		NOS
							Gene	SNP^#^	
Kote-Jarai	2003	UK	Caucasian	PB	PCR-RFLP	271/277	*LEPR*	rs1137100, rs1137101	7
Ribeiro	2004	Portugal	Caucasian	HB	PCR-RFLP	143/118	*LEP*	rs7799039	6
Gade	2006	USA	NA	NA	PCR-SSR	69/137	*LEP*	D7S1875(Microsatellite)	4
							*LEPR*	Exon-3(Microsatellite)	
Wang	2009	USA	Mixed	PB	TaqMan	258/258	*LEP*	rs2167270	9
							*ADIPOQ*	rs1501299	
Moore	2009	Finland	Caucasian	PB	TaqMan	1041/1048	*LEP*	rs7799039, rs2167270, rs1349419, rs12535708, rs12535747, rs791620	9
							*LEPR*	rs1137100, rs1887285, rs7883, rs7602	
							*ADIPOQ*	rs182052, rs822393, rs2241766, rs17366473	
Beebe-Dimmer	2010	USA	African-American	PB	Taqman	131/344	*ADIPOQ*	rs266729, rs822395, rs822396, rs2241766, rs1501299	6
							*ADIPOR1*	rs2232853, rs1342387, rs7539542, rs10920531	
Kaklamani	2011	USA	Mixed	PB	Taqman	465/441	*ADIPOQ*	rs266729, rs822395, rs822396, rs1501299, rs2241766	8
							*ADIPOR1*	rs12733285, rs1342387, rs7539542, rs2232853, rs10920531	
Dhillon	2011	USA	Caucasian	PB	Taqman	1286/1267	*ADIPOQ*	rs266729, rs182052, rs168681205, rs168681209, rs822391, rs12495941, rs17366568, rs3821799, rs3774261, rs17366743, rs2082940, rs7639352	9
							*ADIPOR1*	rs10920531, rs7539542, rs16850799, rs12733285, rs7514221	
							*ADIPOR2*	rs1029629, rs7975600, rs12826079, rs11061946, rs10773983, rs1058322, rs10773986, rs11061973, rs7967137, rs1044471, rs1044825	
Ribeiro	2012	Portugal	Caucasian	HB	TaqMan/PCR-RFLP	449/557	*LEP*	rs7799039	7
							*LEPR*	rs1137100, rs8179183, rs1137101	
Gu	2014	China	Asian	PB	Taqman	917/1036	*ADIPOQ*	rs3774262, rs266729, rs182052	9
Nitta	2016	Japan	NA	NA	NA	198/122	*ADIPOQ*	rs2241766, rs266729, rs822395	4

*NOS* Newcastle-Ottawa Scale, *SNP* single nucleotide polymorphism, *PB* population-based, *HB* hospital-based, *NA* not available, *PCR* polymerase chain reaction, *RFLP* restriction fragment length polymorphism, *SSR* simple sequence repeats

# Common synonyms for genetic polymorphisms: LEP G2548A(rs7799039), LEP A19G(rs2167270), LEPR K109R(rs1137100), LEPR Q223R(rs1137101), LEPR K656N (rs8179183)

**Table 2:** Meta-analysis of multiple studies identifying the associations between *LEP/LEPR/ADIPOQ/ADIPOR1* polymorphisms and prostate cancer risk

Genetic polymorphisms^#^	No. of studies	Dominant model		Recessive model		Homozygote model		Heterozygote model		Allele contrast model	
		OR(95%CI)	P*	OR(95%CI)	P*	OR(95%CI)	P*	OR(95%CI)	P*	OR(95%CI)	P*
*LEP*											
rs7799039[G/A]	3	1.133(0.966-1.328)	0.033	**1.241(1.044-1.474)**	0.47	**1.282(1.043-1.576)**	0.099	1.073(0.907-1.270)	0.049	**1.133(1.024-1.254)**	0.085
rs2167270[G/A]	2	0.898(0.760-1.060)	0.182	0.959(0.752-1.225)	0.672	0.911(0.702-1.183)	0.389	0.896(0.752-1.068)	0.203	0.935(0.828-1.055)	0.233
*LEPR*											
rs1137100[A/G]	3	1.006(0.878-1.154)	0.883	1.065(0.854-1.329)	0.996	1.054(0.834-1.332)	0.977	0.993(0.860-1.147)	0.867	1.018(0.918-1.129)	0.93
rs1137101[A/G]	2	**0.704(0.570-0.871)**	0.207	0.966(0.747-1.248)	0.609	0.774(0.581-1.033)	0.749	**0.680(0.541-0.853)**	0.148	**0.843(0.730-0.973)**	0.641
*ADIPOQ*											
rs266729[C/G]	5	0.959(0.865-1.064)	0.001	1.114(0.913-1.360)	0.511	1.081(0.881-1.327)	0.330	0.937(0.841-1.045)	0.001	0.992(0.913-1.077)	0.007
rs2241766[T/G]	4	**1.249(1.036-1.507)**	0.000	1.055(0.608-1.831)	0.157	1.150(0.654-2.020)	0.347	**1.266(1.044-1.535)**	0.000	**1.201(1.015-1.422)**	0.004
rs1501299[G/T]	3	**0.806(0.669-0.973)**	0.182	0.940(0.689-1.282)	0.956	0.865(0.625-1.197)	0.770	**0.797(0.653-0.972)**	0.166	0.869(0.752-1.004)	0.297
rs182052[G/A]	3	1.045(0.937-1.165)	0.272	1.031(0.907-1.171)	0.042	1.078(0.928-1.253)	0.052	1.03(0.919-1.156)	0.348	1.03(0.958-1.107)	0.111
rs822395[A/C]	3	**0.805(0.652-0.995)**	0.868	1.107(0.805-1.522)	0.250	0.979(0.697-1.377)	0.314	**0.754(0.602-0.944)**	0.810	0.909(0.776-1.065)	0.73
rs822396[A/G]	2	0.901(0.710-1.145)	0.213	**2.187(1.211-3.950)**	0.056	**2.068(1.133-3.774)**	0.093	0.798(0.620-1.026)	0.040	1.023(0.833-1.255)	0.779
*ADIPOR1*											
rs10920531[C/A]	3	1.12(0.975-1.287)	0.565	**1.553(1.280-1.883)**	0.001	**1.66(1.335-2.065)**	0.008	1.01(0.873-1.168)	0.564	**1.184(1.075-1.305)**	0.144
rs7539542[G/C]	3	1.056(0.916-1.219)	0.974	0.914(0.767-1.089)	0.676	1.004(0.809-1.247)	0.997	1.080(0.928-1.256)	0.842	0.998(0.905-1.100)	0.683
rs12733285[C/T]	2	0.985(0.859-1.129)	0.012	0.962(0.776-1.192)	0.007	0.954(0.760-1.196)	0.002	0.986(0.854-1.139)	0.077	0.983(0.888-1.088)	0.002
rs1342387[C/T]	2	0.872(0.678-1.120)	0.536	0.924(0.717-1.192)	0.440	0.859(0.632-1.168)	0.887	0.881(0.674-1.152)	0.341	0.920(0.787-1.076)	0.947
rs2232853[G/A]	2	**0.723(0.571-0.916)**	0.009	**0.378(0.268-0.532)**	0.628	**0.340(0.234-0.494)**	0.528	0.911(0.707-1.173)	0.109	**0.638(0.535-0.760)**	0.002

*OR* odds ratio, *CI* confidence interval

* P value of Q test for assessing heterogeneity.

# Common synonyms for genetic polymorphisms: LEP G2548A(rs7799039), LEP A19G(rs2167270), LEPR K109R(rs1137100), LEPR Q223R(rs1137101)

In the meta-analysis (Table 1), all studies were case-control design. Studies were conducted from 2003 to 2016. Among them, five were performed in USA, another two were performed in Portugal, and the rest four were performed in UK, Finland, China and Japan, respectively. The Newcastle-Ottawa Scale (NOS) scores ranged from 4 to 9. Polymorphisms with meta-analyzed statistics were presented in Table 2, and those with single supporting study were pooled in Table 3. The population number of case and control in each genetic variant and associated forest plots were also displayed (Supplemental Data S1, Supplemental Data S2). Besides, the genetic variants were found to be associated with PCa aggressiveness, e.g. pathological grade, castration resistance, recurrence and metastasis, and survival (Table 4).

**Table 3:** Pooled-review of the studies investigating associations between *LEP/LEPR/ADIPOQ/ADIPOR1/ADIPOR2* polymorphisms and prostate cancer risk

Genetic polymorphisms	Author	Year	Dominant model	Recessive model	Homozygote model	Heterozygote model	Allele contrast model
			OR(95%CI)	OR(95%CI)	OR(95%CI)	OR(95%CI)	OR(95%CI)
*LEP*							
rs1349419[G/A]	Moore	2009	**0.771(0.635-0.936)**	0.930(0.729-1.186)	0.797(0.608-1.044)	**0.762(0.620-0.936)**	**0.865(0.757-0.988)**
rs12535708[C/A]	Moore	2009	**0.818(0.679-0.986)**	0.885(0.668-1.172)	0.804(0.597-1.082)	0.823(0.675-1.003)	**0.869(0.757-0.998)**
rs12535747[C/A]	Moore	2009	**0.808(0.671-0.973)**	0.872(0.660-1.152)	0.789(0.587-1.059)	**0.814(0.668-0.992)**	**0.860(0.749-0.987)**
rs791620[C/A]	Moore	2009	0.906(0.685-1.199)	-	-	0.906(0.685-1.199)	0.912(0.696-1.196)
D7S1875(Microsatellite)[S/L]^#^	Gade	2006	**0.475((0.259-0.872)**	0.705(0.216-2.301)	0.534(0.160-1.778)	**0.464(0.243-0.885)**	**0.573(0.347-0.945)**
*LEPR*							
rs1887285[A/G]	Moore	2009	0.789(0.595-1.046)	2.106(0.646-6.865)	2.031(0.623-6.623)	**0.746(0.558-0.997)**	0.845(0.649-1.102)
rs7883[G/A]	Moore	2009	1.283(0.960-1.714)	4.403(0.513-37.764)	4.515(0.526-38.733)	1.246(0.929-1.670)	1.032(0.989-1.719)
rs7602[G/A]	Moore	2009	0.914(0.730-1.145)	2.260(0.802-6.367)	2.201(0.780-6.205)	0.879(0.699-1.105)	0.962(0.782-1.183)
K656N(rs8179183)[G/C]	Ribeiro	2012	0.836(0.644-1.085)	1.251(0.631-2.479)	1.164(0.584-2.321)	0.807(0.616-1.057)	0.898(0.719-1.122)
Exon-3(Microsatellite)[S/L]^#^	Gade	2006	**1.875(1.030-3.412)**	**5.516(1.857-16.383)**	**6.800(2.170-21.312)**	1.484(0.792-2.780)	**1.918(1.245-2.955)**
*ADIPOQ*							
rs12495941[G/T]	Dhillon	2011	1.008(0.857-1.186)	1.074(0.846-1.363)	1.069(0.828-1.379)	0.991(0.834-1.177)	1.022(0.908-1.151)
rs168681205[G/A]	Dhillon	2011	1.147(0.904-1.456)	0.702(0.222-2.218)	0.716(0.226-2.262)	1.169(0.917-1.490)	1.116(0.890-1.399)
rs168681209[C/A]	Dhillon	2011	0.884(0.721-1.082)	1.178(0.526-2.640)	1.149(0.512-2.577)	0.871(0.708-1.072)	0.907(0.752-1.095)
rs17366568[G/A]	Dhillon	2011	1.116(0.925-1.348)	1.284(0.621-2.655)	1.313(0.634-2.720)	1.106(0.913-1.341)	1.111(0.936-1.319)
rs17366743[T/C]	Moore	2009	1.086(0.737-1.599)	0.300(0.012-7.367)	0.302(0.012-7.413)	1.108(0.751-1.635)	1.061(0.726-1.550)
rs2082940[C/T]	Dhillon	2011	**0.785(0.647-0.954)**	0.526(0.273-1.012)	**0.504(0.262-0.971)**	**0.814(0.666-0.994)**	**0.780(0.654-0.931)**
rs3774261[G/A]	Dhillon	2011	0.969(0.823-1.142)	0.910(0.730-1.133)	0.905(0.711-1.151)	0.991(0.833-1.179)	0.960(0.856-1.077)
rs3774262[G/A]	Gu	2014	0.898(0.752-1.073)	**0.659(0.484-0.898)**	**0.649(0.471-0.895)**	0.966(0.801-1.166)	**0.863(0.752-0.989)**
rs3821799[C/T]	Dhillon	2011	0.898(0.755-1.068)	0.934(0.768-1.136)	0.879(0.701-1.102)	0.906(0.754-1.089)	0.934(0.835-1.045)
rs7639352[C/T]	Dhillon	2011	1.052(0.895-1.237)	1.017(0.757-1.366)	1.039(0.768-1.407)	1.055(0.889-1.252)	1.036(0.911-1.179)
rs822391[T/C]	Dhillon	2011	**0.812(0.687-0.960)**	0.879(0.588-1.315)	0.824(0.549-1.236)	**0.810(0.681-0.965)**	**0.845(0.733-0.975)**
rs822393[C/T]	Moore	2009	0.971(0.801-1.177)	0.825(0.646-1.054)	0.837(0.638-1.098)	1.026(0.835-1.261)	0.931(0.814-1.064)
*ADIPOR1*							
rs16850799[C/T]	Dhillon	2011	0.919(0.780-1.084)	0.949(0.668-1.348)	0.921(0.645-1.315)	0.919(0.773-1.092)	0.936(0.817-1.073)
*ADIPOR2*							
rs1029629[T/G]	Dhillon	2011	0.933(0.795-1.095)	0.975(0.740-1.284)	0.943(0.708-1.256)	0.931(0.786-1.012)	0.955(0.844-1.080)
rs1044471[C/T]	Dhillon	2011	1.013(0.848-1.210)	1.163(0.967-1.398)	1.131(0.907-1.410)	0.957(0.792-1.158)	1.063(0.950-1.189)
rs1044825[T/G]	Dhillon	2011	0.942(0.794-1.116)	1.084(0.880-1.335)	1.026(0.812-1.297)	0.912(0.761-1.092)	0.997(0.889-1.118)
rs1058322[C/T]]	Dhillon	2011	0.945(0.805-1.110)	0.833(0.632-1.097)	0.823(0.618-1.096)	0.975(0.823-1.156)	0.932(0.823-1.055)
rs10773983[G/A]	Dhillon	2011	1.010(0.860-1.187)	1.018(0.776-1.335)	1.022(0.770-1.357)	1.008(0.852-1.193)	1.010(0.894-1.140)
rs10773986[A/G]	Dhillon	2011	0.934(0.795-1.097)	1.057(0.810-1.380)	1.014(0.768-1.339)	0.916(0.772-1.085)	0.972(0.860-1.100)
rs11061946[C/T]	Dhillon	2011	1.163(0.926-1.461)	0.614(0.200-1.882)	0.629(0.205-1.928)	1.191(0.944-1.502)	1.123(0.905-1.393)
rs11061973[G/A]	Dhillon	2011	1.041(0.872-1.244)	0.976(0.579-1.643)	0.987(0.585-1.666)	1.046(0.871-1.257)	1.030(0.880-1.205)
rs12826079[C/T]	Dhillon	2011	1.006(0.797-1.271)	1.650(0.598-4.555)	1.647(0.596-4.547)	0.982(0.773-1.246)	1.031(0.827-1.284)
rs7967137[T/C]	Dhillon	2011	0.961(0.794-1.163)	0.976(0.514-1.853)	0.968(0.510-1.841)	0.961(0.789-1.169)	0.966(0.812-1.149)
rs7975600[A/T]	Dhillon	2011	0.884(0.738-1.060)	0.915(0.553-1.516)	0.889(0.536-1.475)	0.884(0.733-1.067)	0.900(0.766-1.056)

*OR* odds ratio, *CI* confidence interval

# S stands for short allele, and L stands for long allele.

**Table 4:** Pooled-review of the studies investigating associations between genetic variants and prostate cancer aggressiveness

Genetic polymorphisms^#^	Author	Year	Associations of minor allele of polymorphisms with PCa aggressiveness (unless otherwise stated)
*LEP*			
rs7799039[G/A]	Ribeiro	2004	Over represented in PCa patients with advanced disease (OR 1.91, 95%CI 1.24-2.59).
rs10244329[A/T]	Reese	2010	Associated with PCa recurrence after definitive treatment (HR 0.49, 95%CI 0.28-0.84, P=0.010).
*LEPR*			
rs8179183[G/C]	Monteiro	2010	Associated with higher Gleason score (P=0.008).
rs1137100[A/G]	Monteiro	2010	Lys carrier had lower time-to-bone metastasis in multivariate analysis (HR 0.37, 95% CI 0.14-0.95, P=0.039).
	Lin	2011	Associated with a decrease in PCa specific mortality (HR 0.82, 95%CI 0.67-1.00, P=0.027) in the Swedish cohort.
	Kapustina	2014	Distinguish PCa patients with high grade (Gleason score ≥7) or low grade (Gleason score <7) cancer.
rs1137101[A/G]	Monteiro	2009	Associated with a higher Gleason score (P=0.022) and shorter time-to-relapse (P=0.006).
	Ribeiro	2012	In multivariate model, AA was associated with high-grade PCa (Gleason score ≥7) (OR 1.56, 95%CI 1.15-2.12).
*ADIPOQ*			
rs1501299[G/T]	Cunha	2010	Associated with increased risk for higher Gleason score (OR 1.99, 95%CI 1.2-3.3, P=0.004), shorter time to hormonal castration resistance (TT vs. G, P=0.006).
rs182052[G/A]	Gu	2015	Associated with increased risk of biochemical recurrence (AA vs GG, HR 2.44, 95%CI 1.57-3.79, P=6×10^-5^).
*ADIPOR2*			
rs1044471[C/T]	Stark	2011	Associated with time to lethal PCa (CT vs CC, HR 0.6, 95%CI 0.4-0.9; TT vs CC, HR 0.8, 95%CI 0.6-1.2).

*OR* odds ratio, *CI* confidence interval, *PCa* prostate cancer

# Common synonyms for genetic polymorphisms: LEP G2548A(rs7799039), LEPR K656N (rs8179183), LEPR K109R(rs1137100), LEPR Q223R(rs1137101)

### LEP polymorphisms and PCa

**Figure 2:**
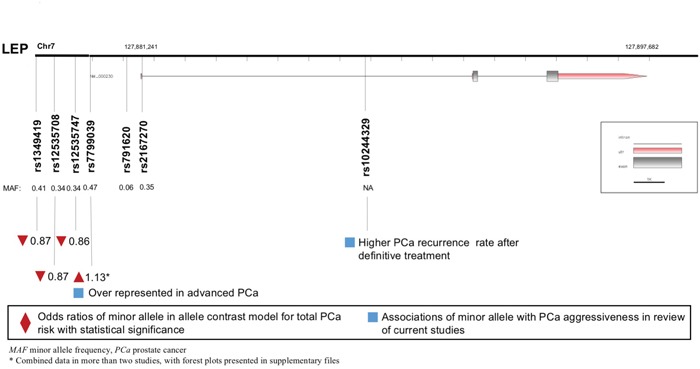
Diagram of *LEP* polymorphisms and prostate cancer

A total of eight *LEP* polymorphisms were enrolled, with key information outlined in Figure 2. *LEP* rs7799039 A allele was correlated with higher risk of PCa (allele contrast: OR 1.133, 95%CI 1.024-1.254, Table 2), which was also over represented in advanced diseases (Table 4). On the contrary, the variants of *LEP* rs1349419 (allele contrast: OR 0.865, 95%CI 0.757-0.988), *LEP* rs12535708 (allele contrast: OR 0.869, 95%CI 0.757-0.998), *LEP* rs12535747 (allele contrast: OR 0.860, 95%CI 0.749-0.987) and *LEP* D7S1875 (allele contrast: OR 0.573, 95%CI 0.347-0.945) were associated with decreased risk of total PCa (Table 3). Besides, *LEP* rs10244329 variant was associated with lower recurrence rate after definitive treatment (Table 4).

### LEPR polymorphisms and PCa

**Figure 3:**
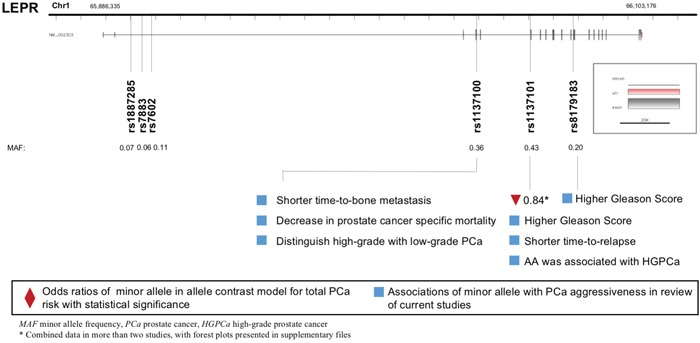
Diagram of *LEPR* polymorphisms and prostate cancer

A total of eight *LEPR* polymorphisms were enrolled, with key data displayed in Figure 3. *LEPR* rs1137101 G allele was associated with lower PCa risk (allele contrast: OR 0.843, 95%CI 0.730-0.973, Table 2), while it was proved to be associated with worse pathological grade and prognosis (Table 4). *LEPR* Exon-3 long allele variant was proved to increase PCa risk (allele contrast: OR 1.918, 95%CI 1.245-2.955, Table 3). Besides, *LEPR* rs8179183 variant was associated with higher Gleason score, and *LEPR* rs1137100 polymorphism was related to both pathological grade and patient survival (Table 4).

### ADIPOQ polymorphisms and PCa

**Figure 4:**
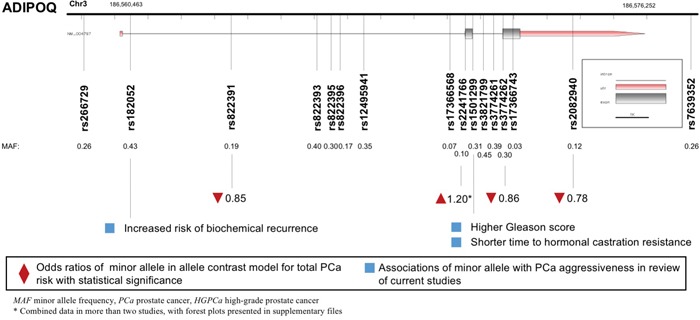
Diagram of *ADIPOQ* polymorphisms and prostate cancer

A total of 18 polymorphisms in gene *ADIPOQ* were enrolled, with key data displayed in Figure 4. *ADIPOQ* rs2241766 G allele was correlated with higher PCa risk (allele contrast: OR 1.201, 95%CI 1.015-1.422, Table 2). On the contrary, the variants of *ADIPOQ* rs2082940 (allele contrast: OR 0.780, 95%CI 0.654-0.931), *ADIPOQ* rs3774262 (allele contrast: OR 0.863, 95%CI 0.752-0.989) and *ADIPOQ* rs822391 (allele contrast: OR 0.845, 95%CI 0.733-0.975) were negatively associated with cancer risk (Table 3). *ADIPOQ* rs1501299 variant was reported to be associated with higher Gleason score and shorter time to castration resistance of PCa (Table 4). Besides, *ADIPOQ* rs182052 variant was associated with increased biochemical recurrence of PCa (Table 4).

### ADIPOR1/ADIPOR2 polymorphisms and PCa

**Figure 5:**
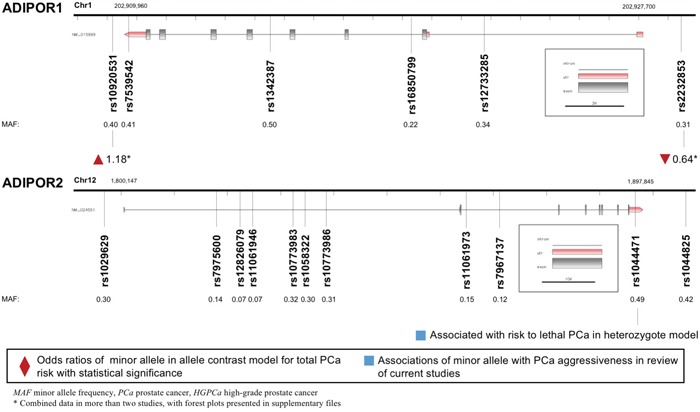
Diagram of *ADIPOR1*/*ADIPOR2* polymorphisms and prostate cancer

Both *ADIPOR1* and *ADIPOR2* encoded proteins that served as receptors for adiponectin. A total of six variants in gene *ADIPOR1* and 11 variants in gene *ADIPOR2* were enrolled and displayed in Figure 5. *ADIPOR1* rs10920531 variant was correlated with higher PCa risk (allele contrast: OR 1.184, 95%CI 1.075-1.305, Table 2). However, a negative association was witnessed between *ADIPOR1* rs2232853 variant and cancer risk (allele contrast: OR 0.638, 95%CI 0.535-0.760, Table 2). We failed to detect any associations between *ADIPOR2* gene polymorphisms and risk of PCa. Meanwhile, *ADIPOR2* rs1044471 variant was negatively associated with time to lethal PCa.

## DISCUSSION

Various clinical and molecular studies were conducted to deliberate the associations among leptin, adiponectin and PCa, while controversies still existed and exact mechanisms remained largely unknown. From a genetic perspective, the present study conducted an updated meta-analysis and pooled-review. We enrolled 49 genetic variants in gene *LEP*, *LEPR, ADIPOQ, ADIPOR1* and *ADIPOR2*, to investigate the relationships between these polymorphisms and PCa. The meta-analysis yielded five polymorphisms that associated with PCa risk according to allele contrast model. Meanwhile, the pooled-review outlined eight variants that affected cancer risk and another eight polymorphisms that affected cancer aggressiveness.

The *LEP* gene, encoded leptin that was associated with obesity and carcinogenesis [13]. In present study, we discovered that minor allele in *LEP* rs7799039 (G2548A) increased both the prevalence and aggressiveness of PCa. The increasing trend in cancer risk was in accordance with previous studies [37, 38]. The rs7799039 was located at promoter sequence of gene *LEP*. Several studies demonstrated that the variants might elevate the serum leptin level through transcriptional level, and increase human body weight as well [39, 40]. Higher leptin level, in turn, contributed to cancer growth and development via stimulating inflammation, angiogenesis, proliferation and epithelial-mesenchymal transition [10, 41].

Leptin exerted its downstream functioning through binding to leptin receptor (encoded by gene *LEPR*). Like *LEP*, *LEPR* was also highly polymorphic, with a number of single nucleotide polymorphisms identified. The present study revealed that *LEPR* rs1137101 (Q223R) variants were correlated with decreased PCa risk, whereas worse pathological grade and prognosis. The previous meta-analysis discovered that rs1137101 might increase the susceptibility to cancers in Asian and African patients, while decreasing the susceptibility in Caucasians, suggesting that race might explain for the heterogeneity and inconsistency among different studies [42]. On the other hand, the associations between rs1137101 and different cancers remained inconsistent [37, 42]. In the present study, the enrolled population were all Caucasians. Whether the negative associations with cancer risk contributed to Caucasian race or cancer type required further validation. It was well studied that rs1137101 mutation (A to G transversion) encoded a glutamine to arginine substitution in the N-terminal CRH1 domain [43], which was proved dispensable for a high affinity interaction with leptin binding [44]. However, the mutation might alter downstream signaling (e.g. STAT3, JAK2), surface expression, or receptor trafficking [44–46], which might partly underlie the mechanisms for worse pathological grade and prognosis of PCa in the present study.

*ADIPOQ* gene was also highly polymorphic, with at least 620 reported polymorphisms. Some of the polymorphisms were studied for their associations with cancer risk [47]. In the present study, we demonstrated that *ADIPOQ* rs2241766 variant was associated with increased risk of PCa. Of note, the outcome was opposite to those found in colorectal cancer, where the variant allele was identified as a protective factor against cancer [14, 47]. The discrepancy remained unknown. To our knowledge, the rs2241766 variant, a synonymous polymorphism at exon 2, might alter mRNA levels by regulation of mRNA splicing or stability [48]. Recently, a meta-analysis discovered that G allele of rs2241766 increased the risk of metabolic syndrome [49]. Meanwhile, metabolic syndrome was closely associated with cancer development and progression [50], which provided a probable mechanism underlying the present outcomes.

Adiponectin receptor 1 and 2 were often differentially expressed in cancers [14]. In the present study, we found no correlation among *ADIPOR1* rs7539542, rs12733285, rs1342387 and risk of PCa, which was in line with the previous study [17]. Of note, we discovered that *ADIPOR1* rs10920531 polymorphism was associated with increased risk of PCa, while *ADIPOR1* rs2232853 variant decreased the risk of PCa. These two polymorphisms were firstly reported that were associated with cancer risk. Since the limitation of related studies, the outcomes required more validation and the underlying mechanisms demand further exploration.

Several limitations existed in the present study. First, the number of studies addressing each polymorphism was limited, and therefore confined us from conducting more meaningful subgroup analyses, e.g. race. Second, the baseline body mass index, serum leptin and adiponectin level were not reported in each study, which might bias the outcomes and constrain us to draw more conclusions. Consequently, more well designed studies with different population were warranted to better identify these complexities.

In conclusion, the present study comprehensively summarized the associations of genetic polymorphisms in *LEP*, *LEPR*, *ADIPOQ*, *ADIPOR1* and *ADIPOR2* with the risk and aggressiveness of PCa. We discovered that *LEP* rs7799039, *ADIPOQ* rs2241766 and *ADIPOR1* rs10920531 variants were correlated with increased risk of PCa, while *LEPR* rs1137101 and *ADIPOR1* rs2232853 variants were associated with decreased risk of PCa in the meta-analysis. From the pooled-review, we further identified eight variants associated with cancer risk and another eight variants associated with cancer aggressiveness, respectively. These observations provided rich evidence that leptin and adiponectin, along with their receptors, were involved in the development and progression of PCa. We expected to help prediction of prevalence and prognosis of PCa, as well as generation of novel targeted therapy, especially in obese patients with impaired leptin and adiponectin signaling.

## MATERIALS AND METHODS

### Search strategy

We conducted a comprehensive literature search in PubMed and EMBASE database up to May 1^st^, 2016. Searching strategy involved “prostatic neoplasms OR prostatic cancer OR prostate cancer OR PCa”, “polymorphism* OR variant* OR SNP”, “leptin OR *LE*P OR *LEPR* OR adiponectin OR *ADIPOQ* OR ADIPOR*” and various combinations of these terms. The search was conducted without limitations of language. Reviews, meta-analyses, original articles, and other studies of interest were further examined to identify additional eligible studies.

### Selection criteria

The eligible studies were included according to the following criteria: (1) studies investigating the associations between *LEP*/*LEPR*/*ADIPOQ*/*ADIPOR1*/*ADIPOR2* polymorphisms and PCa risk or aggressiveness; (2) case-control or cohort studies; (3) sufficient data, including the accurate population number of specific alleles, genotypes and microsatellites; (4) genotype frequencies in control groups should be examined by the Hardy-Weinberg equilibrium (HWE). If serial studies of the same study population were reported, the most recent study or the study with the largest population should be adopted. The exclusion criteria included: (1) animal or molecular studies; (2) reviews, meta-analyses or case reports; (3) insufficient information published in the study; (4) duplicated studies.

### Data extraction and quality assessment

Two investigators (Hu and Xu) independently evaluated the eligibility of all retrieved studies, and extracted key information. The collected data included: author's name, year of publication, study population, study design, genotyping methods, case and control population, and polymorphisms investigated. NOS was adopted to evaluate the quality of all eligible studies [51]. The nine-star NOS was based on three dimensions: study group selection, comparability of cases and controls, and exposure of cases and controls. The disagreements during data extraction and quality assessment were resolved by consensus.

### Meta-analysis and pooled review

In the meta-analysis, the pooled odds ratio (OR) with 95% confidence interval (95%CI) was calculated to evaluate the correlation between genetic polymorphisms and total PCa risk according to allele contrast, homozygote, heterozygote, dominant and recessive models. The inter-study heterogeneity was verified by Q and I^2^ statistics. Fixed-effect model was adopted to estimate the OR and 95%CI when heterogeneity was low, otherwise random-effect model was adopted. Besides, we conducted a pooled-review for studies discussing risk or aggressiveness of PCa that could not be included in the meta-analysis. All analyses were performed using the STATA/SE 12.0 software (StataCorp, College Station, TX, USA). Statistical significance was defined as a two-tailed p-value <0.05.

## SUPPLEMENTARY MATERIALS FIGURES AND TABLES




