# Supplementary material for: Genetic polymorphisms in leptin, adiponectin and their receptors affect risk and aggressiveness of prostate cancer: evidence from a meta-analysis and pooled-review
Source: Oncotarget. 2016 Oct 19;7(49):81049–61. doi: 10.18632/oncotarget.12747 (PMC5348375; doi:10.18632/oncotarget.12747)

Genetic polymorphisms in leptin, adiponectin and their receptors affect risk and aggressiveness of prostate cancer: evidence from a meta-analysis and pooled-review

SUPPLEMENTARY DATA

Supplementary Data S1: Raw data of population of each genetic variant in the meta-analysis

See Supplementary File 1

Supplemental Data S2: Forest plots of allele contrast model demonstrating associations between different genetic polymorphisms and prostate cancer risk

LEP G2548A (rs7799039) [G/A]
A vs G

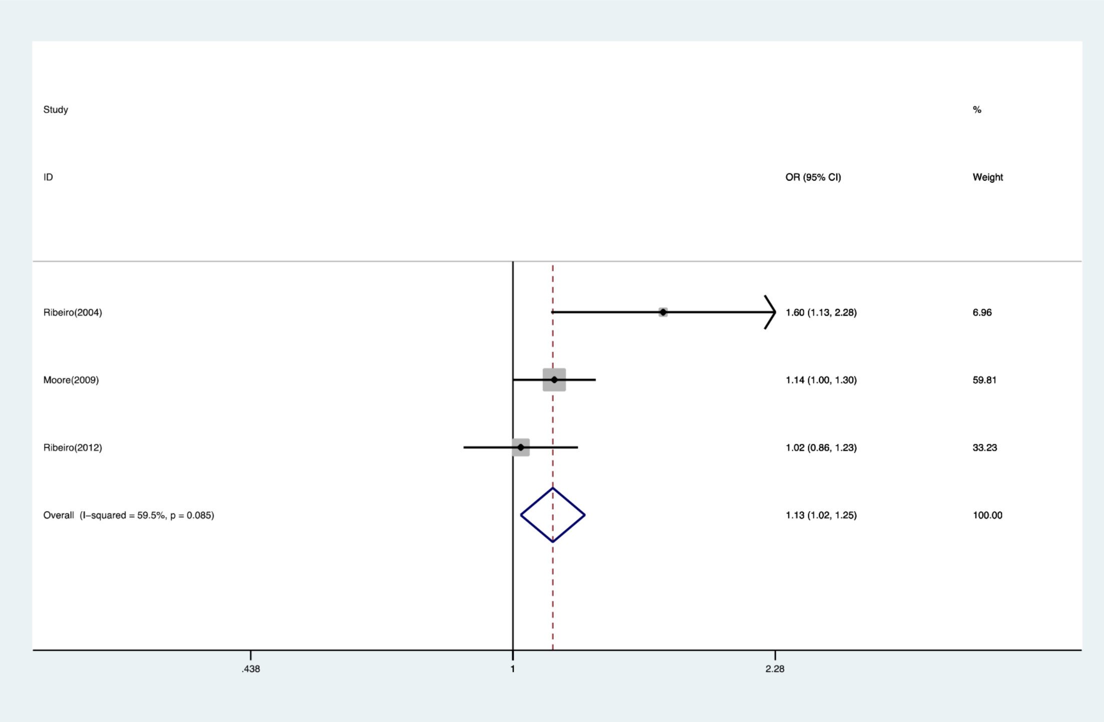

LEP A19G (rs2167270) [G/A]
A vs G

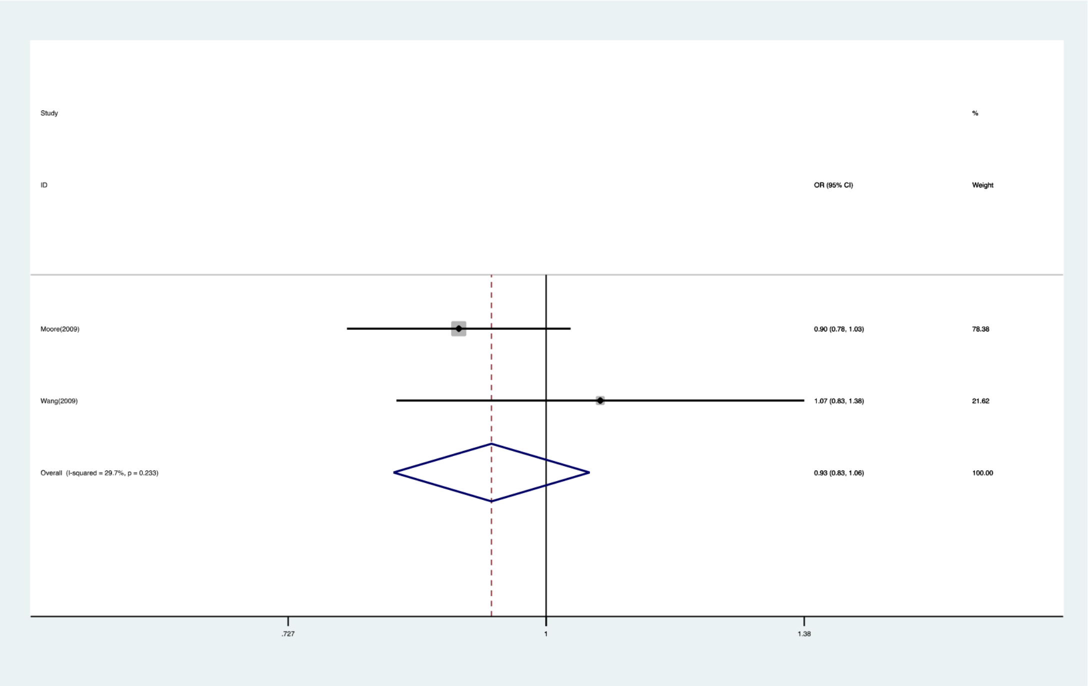

LEPR K109R (rs1137100) [A/G]
G vs A

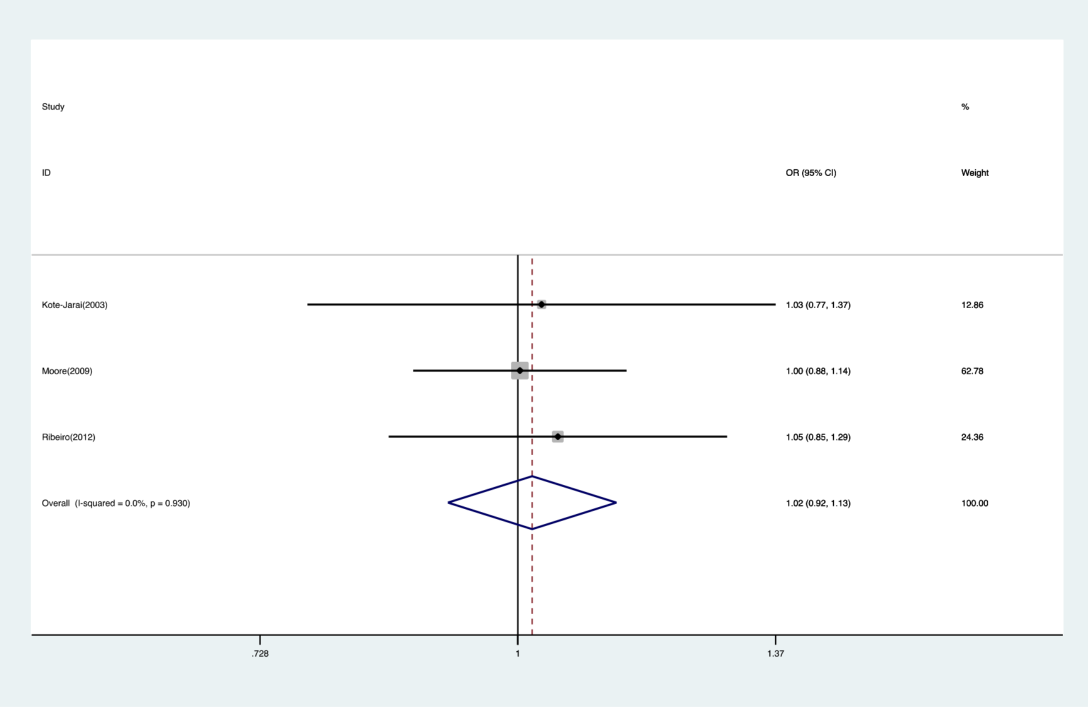

LEPR Q223R (rs1137101) [A/G]
G vs A

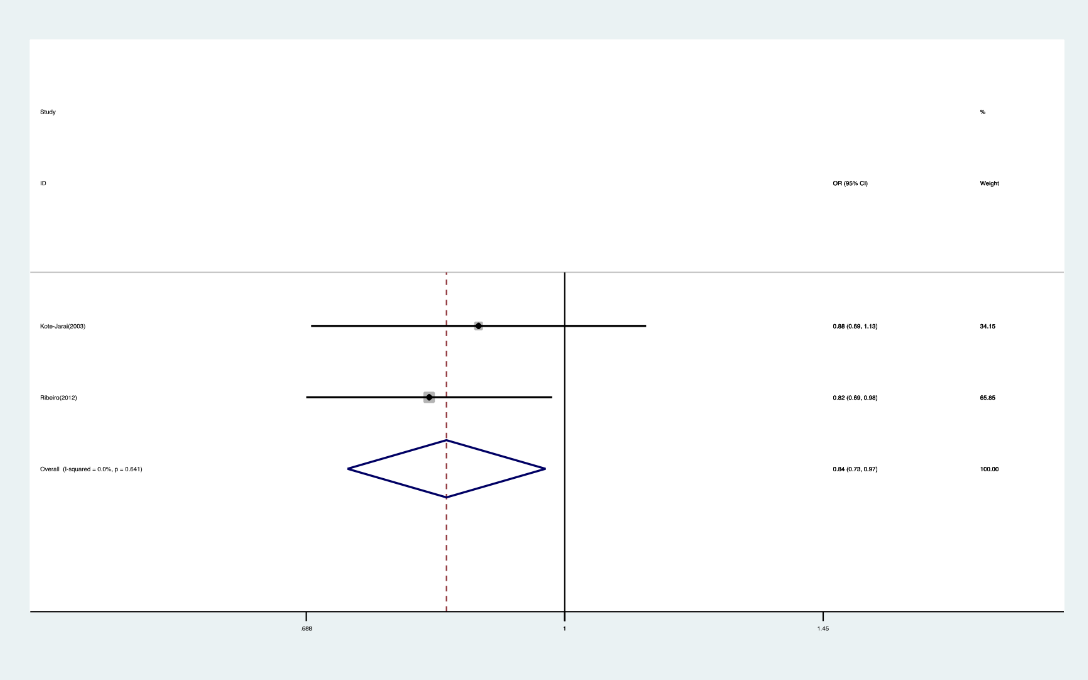

ADIPOQ rs266729 [C/G]
G vs C

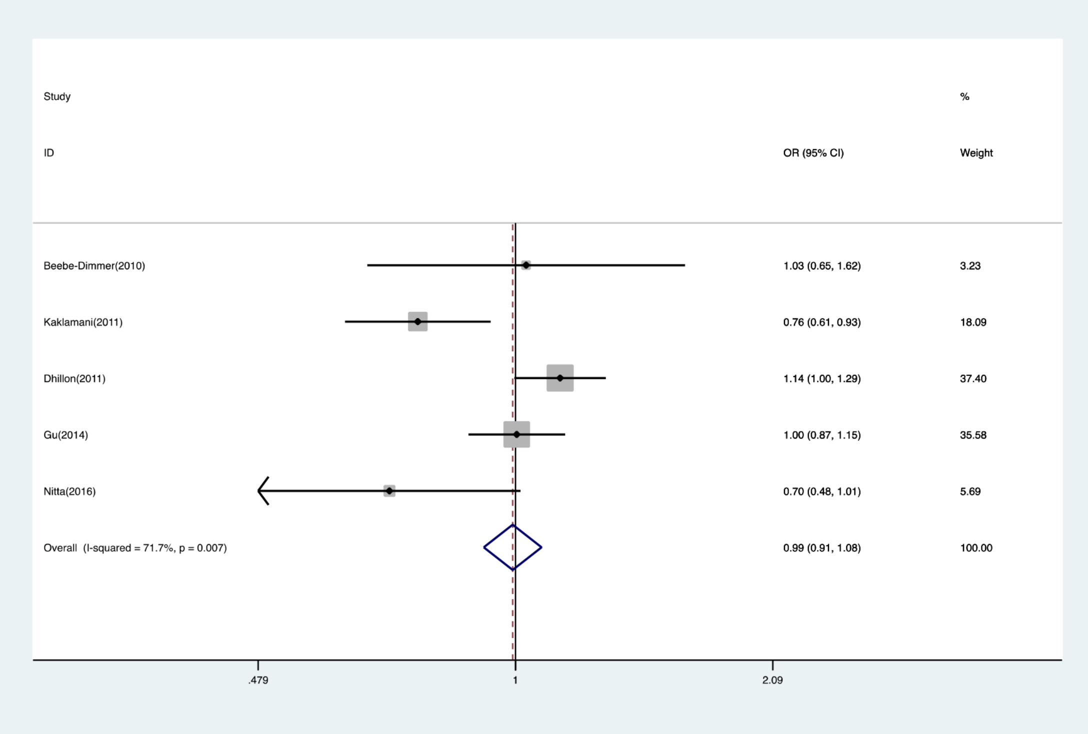

ADIPOQ rs2241766 [T/G]
G vs T

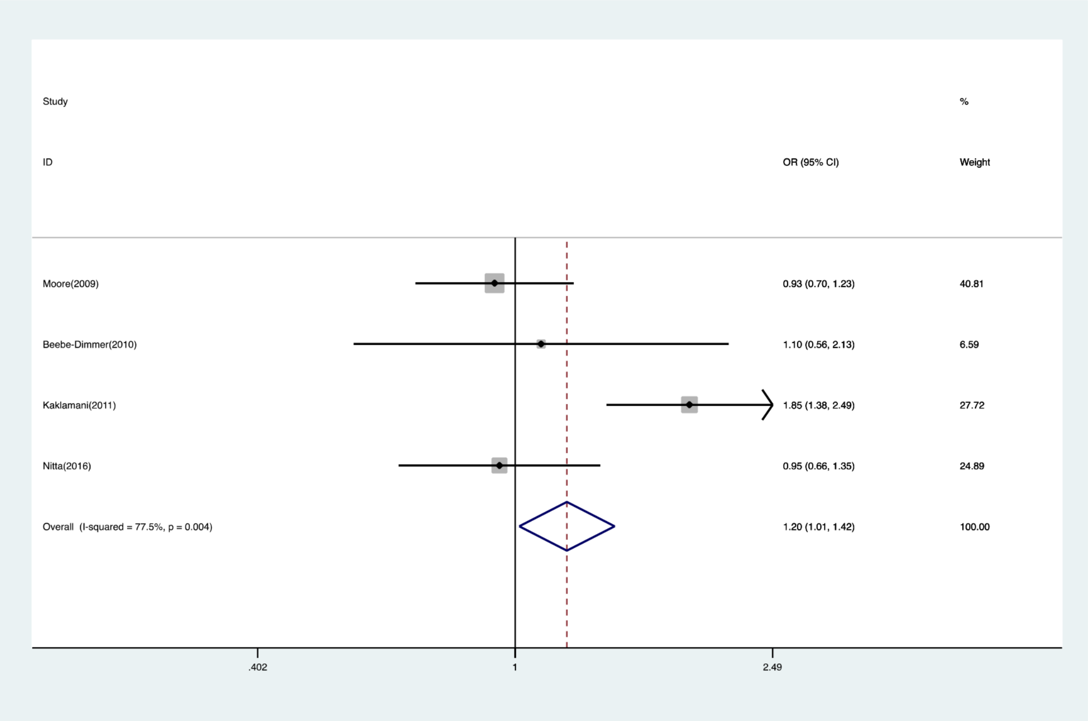

ADIPOQ rs1501299 [G/T]
T vs G

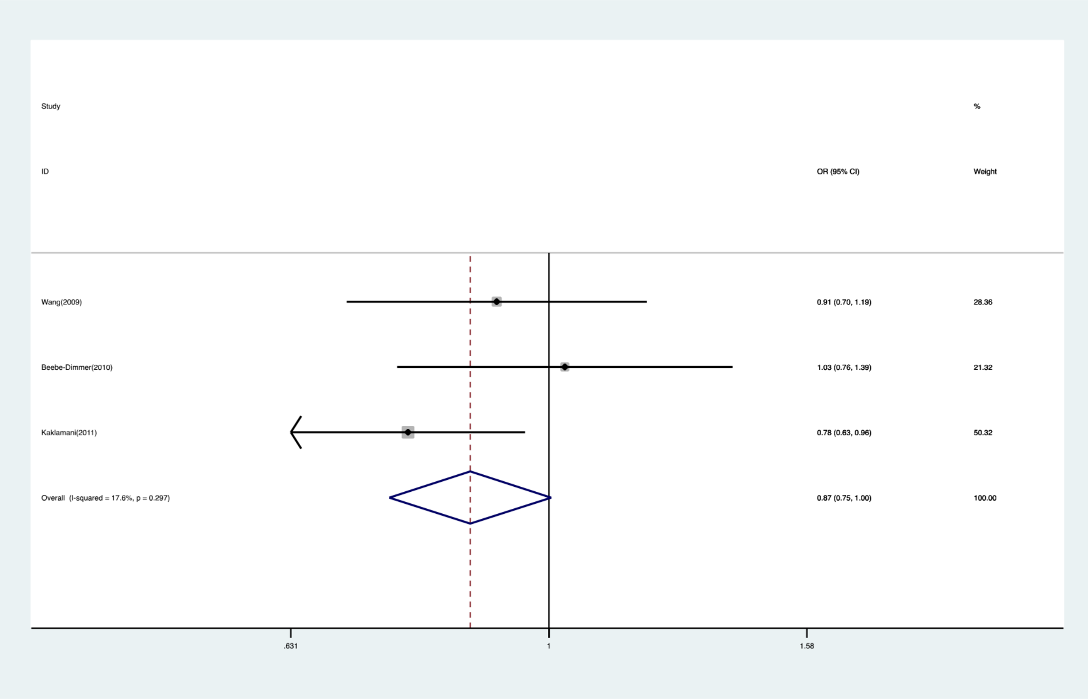

ADIPOQ rs182052 [G/A]
A vs G

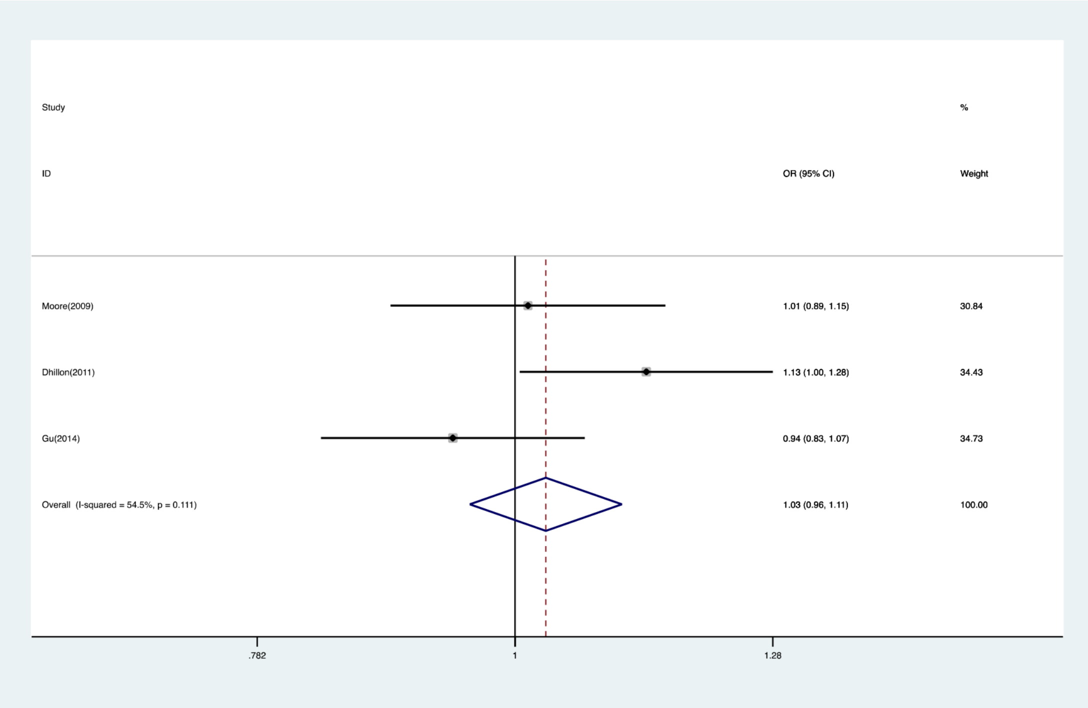

ADIPOQ rs822395 [A/C]
C vs A

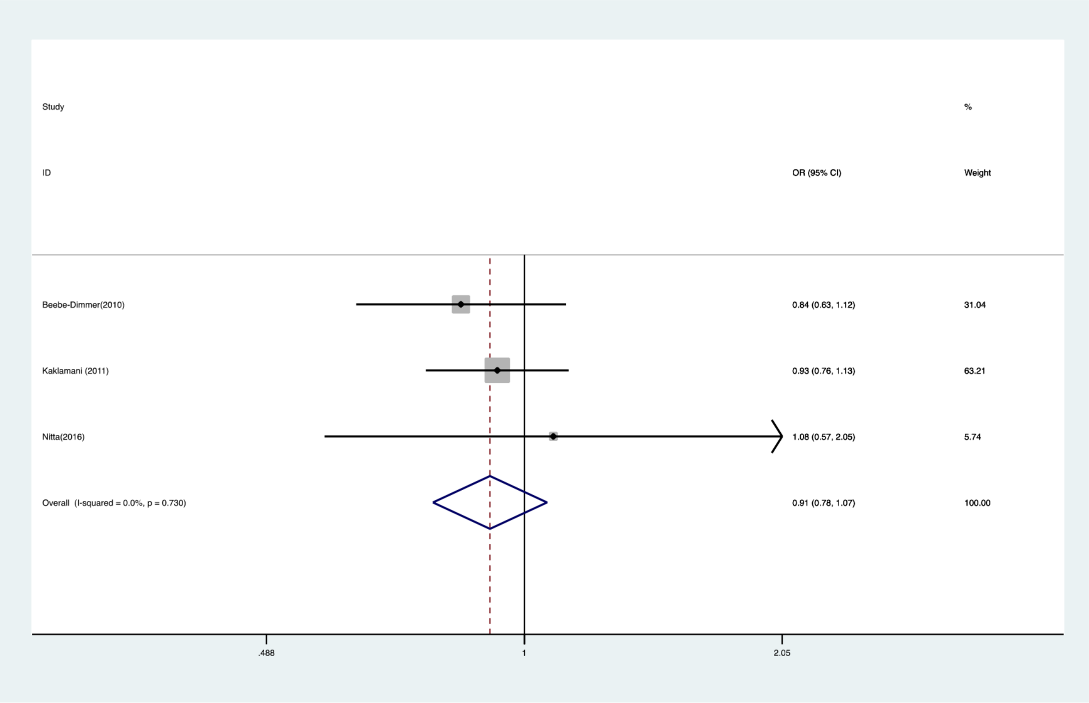

ADIPOQ rs822396 [A/G]
G vs A

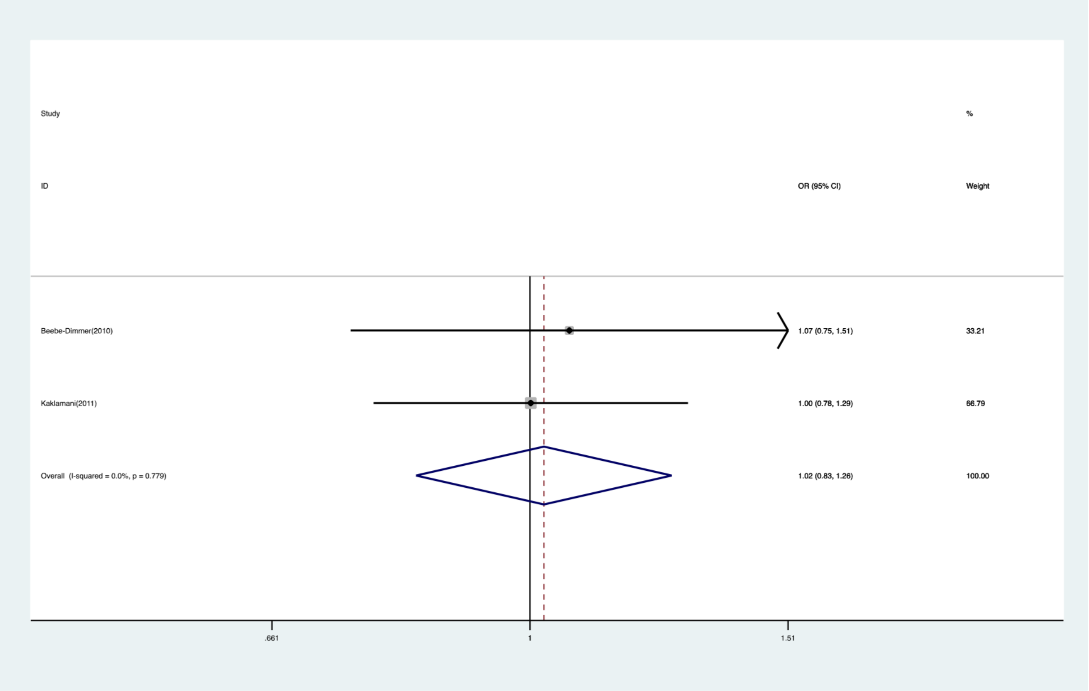

***ADIPOR1* rs10920531 [C/A]**
A vs C

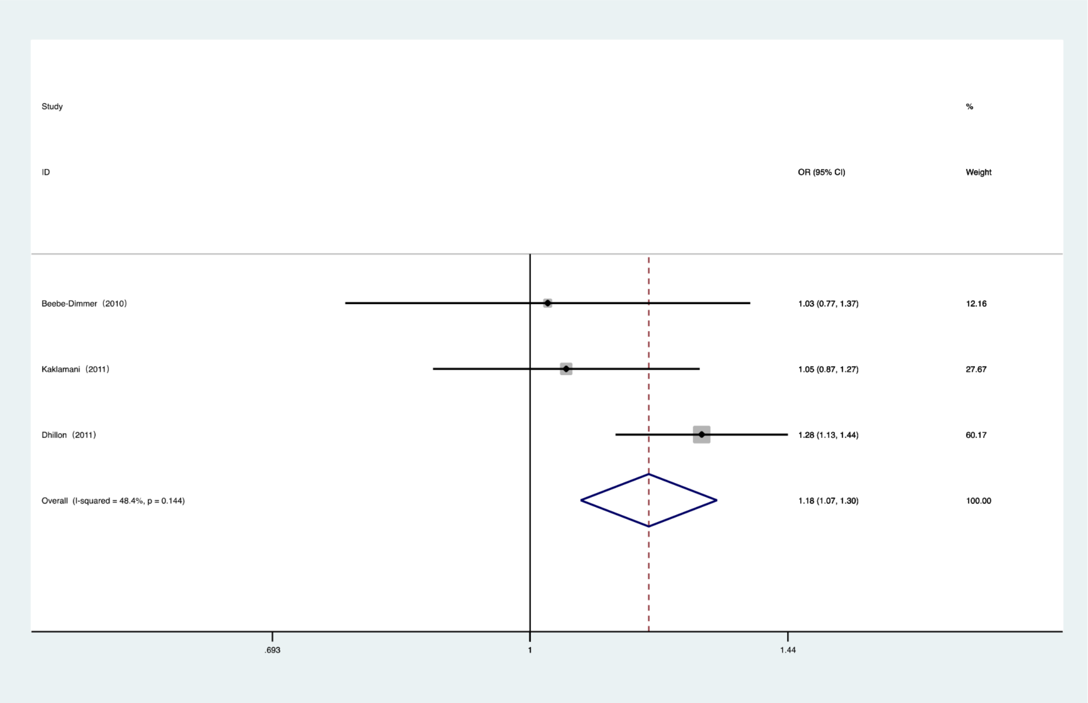

***ADIPOR1* rs7539542 [G/C]**
C vs G

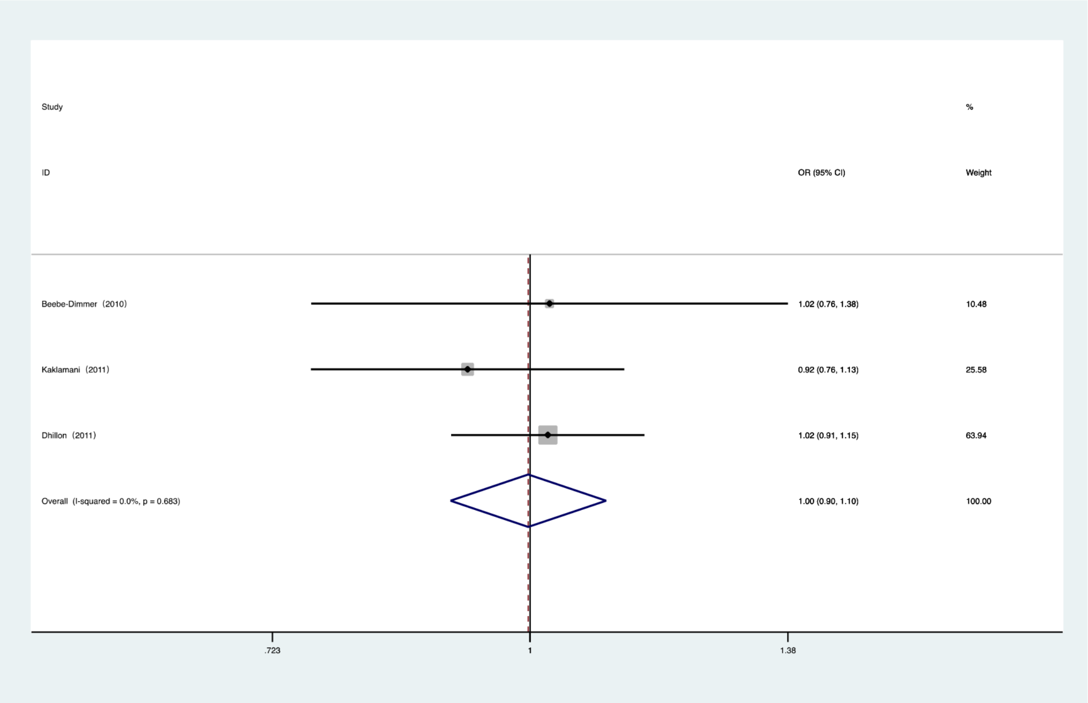

***ADIPOR1* rs12733285 [C/T]**
T vs C

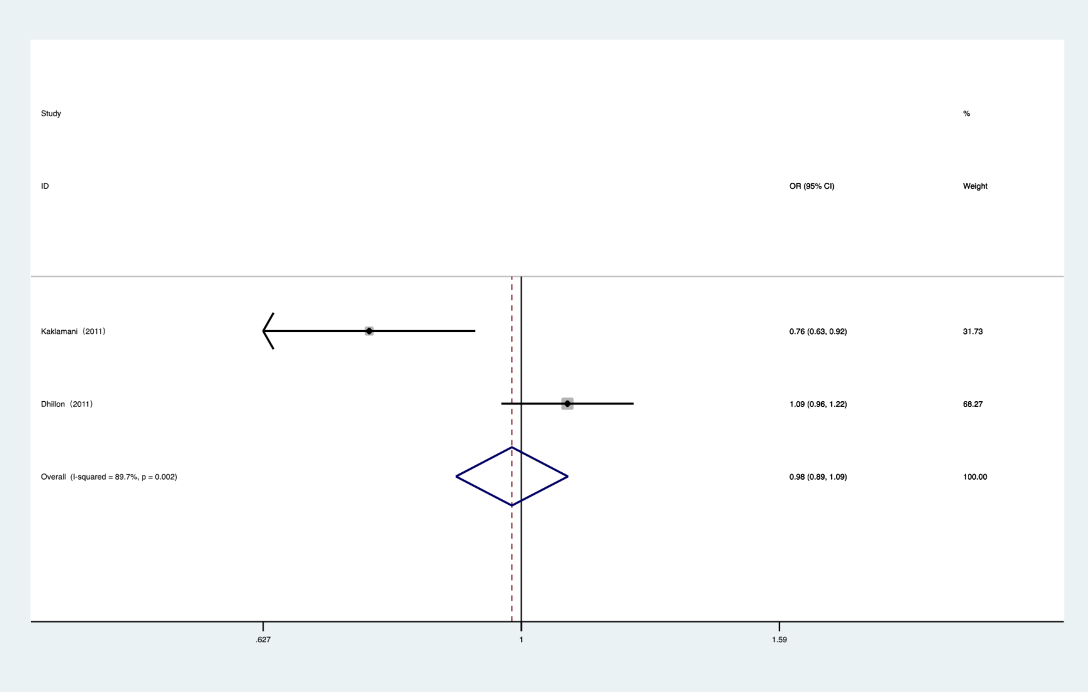

***ADIPOR1* rs1342387 [C/T]**
T vs C

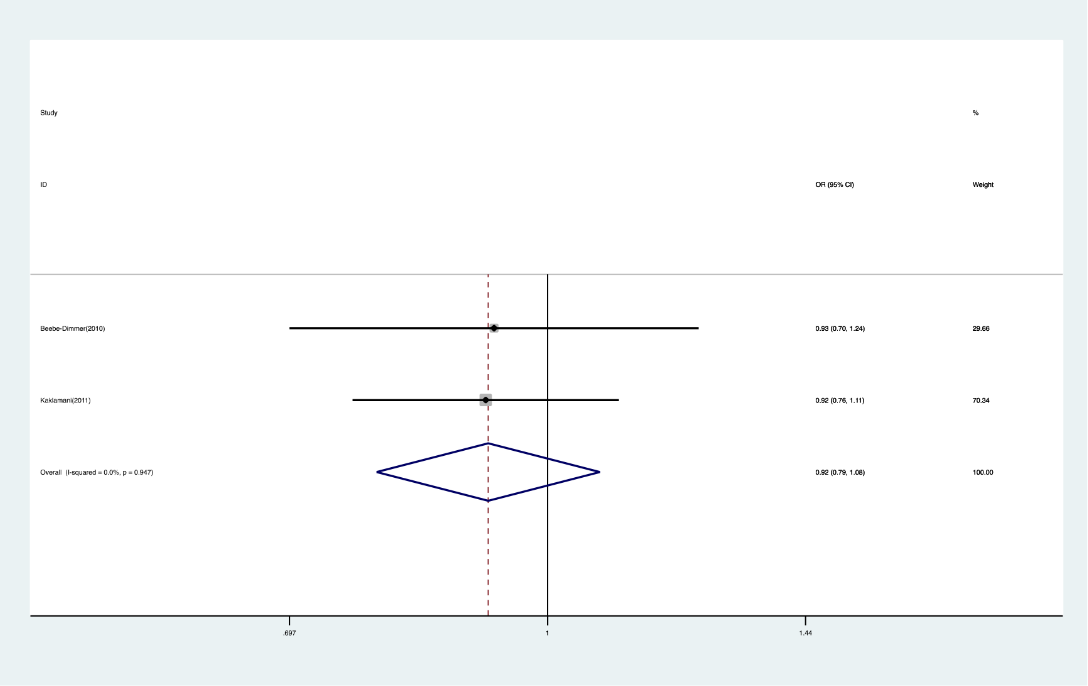

ADIPOR1 **rs2232853 [G/A]**
A vs G

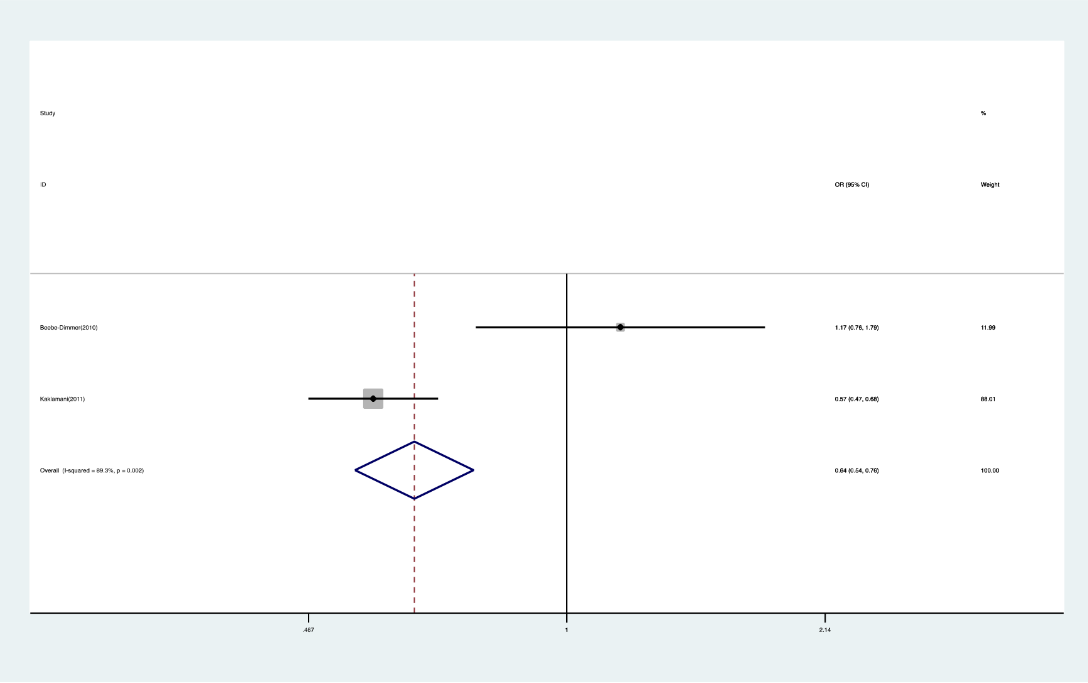

Supplement: Supplementary file 1 [file oncotarget-07-81049-s001.pdf]
