# Supplementary material for: Genetic polymorphisms in leptin, adiponectin and their receptors affect risk and aggressiveness of prostate cancer: evidence from a meta-analysis and pooled-review
Source: Oncotarget. 2016 Oct 19;7(49):81049–61. doi: 10.18632/oncotarget.12747 (PMC5348375; doi:10.18632/oncotarget.12747)
Supplement: Supplementary file 2 [file oncotarget-07-81049-s002.docx]

**Supplemental Data S1** Raw data of population of each genetic variant in the meta-analysis

| Study | Year | Case | | | Control | | |
| --- | --- | --- | --- | --- | --- | --- | --- |
| *LEP G2548A(rs7799039)* |  | GG | GA | AA | GG | GA | AA |
| Ribeiro | 2004 | 30 | 89 | 24 | 44 | 62 | 12 |
| Moore | 2009 | 213 | 453 | 281 | 210 | 437 | 216 |
| Ribeiro | 2012 | 164 | 212 | 73 | 203 | 268 | 84 |
| *LEP A19G(rs2167270)* |  | GG | GA | AA | GG | GA | AA |
| Moore | 2009 | 428 | 404 | 113 | 346 | 387 | 107 |
| Wang | 2009 | 92 | 122 | 39 | 100 | 119 | 38 |
| *LEP rs1349419* |  | GG | GA | AA | GG | GA | AA |
| Moore | 2009 | 365 | 419 | 161 | 280 | 422 | 155 |
| *LEP rs12535708* |  | CC | CA | AA | CC | CA | AA |
| Moore | 2009 | 443 | 390 | 111 | 356 | 381 | 111 |
| *LEP rs12535747* |  | CC | CA | AA | CC | CA | AA |
| Moore | 2009 | 447 | 387 | 112 | 362 | 385 | 115 |
| *LEP rs791620* |  | CC | CA | AA | CC | CA | AA |
| Moore | 2009 | 829 | 113 | 0 | 738 | 111 | 0 |
| *LEP D7S1875(Microsatellite) ^#^* |  | SS | SL | LL | SS | SL | LL |
| Gade | 2006 | 47 | 18 | 4 | 69 | 57 | 11 |
| *LEPR K109R(rs1137100)* |  | AA | AG | GG | AA | AG | GG |
| Kote-Jarai | 2003 | 131 | 87 | 18 | 140 | 91 | 18 |
| Moore | 2009 | 415 | 462 | 140 | 369 | 428 | 120 |
| Ribeiro | 2012 | 264 | 157 | 28 | 334 | 190 | 32 |
| *LEPR Q223R(rs1137101)* |  | AA | AG | GG | AA | AG | GG |
| Kote-Jarai | 2003 | 109 | 106 | 58 | 94 | 107 | 61 |
| Ribeiro | 2012 | 166 | 206 | 77 | 151 | 312 | 94 |
| *LEPR rs1887285* |  | AA | AG | GG | AA | AG | GG |
| Moore | 2009 | 780 | 95 | 9 | 704 | 115 | 4 |
| *LEPR rs7883* |  | GG | GA | AA | GG | GA | AA |
| Moore | 2009 | 835 | 120 | 5 | 754 | 87 | 1 |
| *LEPR rs7602* |  | GG | GA | AA | GG | GA | AA |
| Moore | 2009 | 768 | 189 | 13 | 650 | 182 | 5 |
| *K656N(rs8179183)* |  | GG | GC | CC | GG | GC | CC |
| Ribeiro | 2012 | 298 | 133 | 17 | 347 | 192 | 17 |
| *LEPR Exon-3(Microsatellite)^#^* |  | SS | SL | LL | SS | SL | LL |
| Gade | 2006 | 24 | 33 | 12 | 68 | 63 | 5 |
| *ADIPOQ rs266729* |  | CC | CG | GG | CC | CG | GG |
| Beebe-Dimmer | 2010 | 104 | 24 | 3 | 248 | 62 | 4 |
| Kaklamani | 2011 | 268 | 153 | 36 | 204 | 208 | 29 |
| Dhillon | 2011 | 636 | 477 | 79 | 675 | 443 | 65 |
| Gu | 2014 | 470 | 358 | 89 | 527 | 414 | 95 |
| Nitta | 2016 | 127 | 62 | 9 | 67 | 45 | 10 |
| *ADIPOQ rs2241766* |  | TT | TG | GG | TT | TG | GG |
| Moore | 2009 | 842 | 105 | 0 | 757 | 102 | 0 |
| Beebe-Dimmer | 2010 | 114 | 11 | 1 | 297 | 31 | 0 |
| Kaklamani | 2011 | 296 | 136 | 15 | 280 | 42 | 16 |
| Nitta | 2016 | 108 | 74 | 16 | 60 | 56 | 6 |
| *ADIPOQ rs1501299* |  | GG | GT | TT | GG | GT | TT |
| Wang | 2009 | 131 | 94 | 28 | 121 | 104 | 28 |
| Beebe-Dimmer | 2010 | 53 | 61 | 17 | 137 | 142 | 44 |
| Kaklamani | 2011 | 261 | 160 | 36 | 203 | 184 | 37 |
| *ADIPOQ rs182052* |  | AA | AG | GG | AA | AG | GG |
| Moore | 2009 | 266 | 472 | 205 | 252 | 400 | 202 |
| Dhillon | 2011 | 147 | 527 | 545 | 108 | 524 | 564 |
| Gu | 2014 | 205 | 448 | 264 | 243 | 514 | 279 |
| *ADIPOQ rs822395* |  | AA | AC | CC | AA | AC | CC |
| Beebe-Dimmer | 2010 | 50 | 61 | 20 | 112 | 165 | 63 |
| Kaklamani | 2011 | 229 | 165 | 61 | 194 | 196 | 47 |
| Nitta | 2016 | 173 | 22 | 3 | 106 | 16 | 0 |
| *ADIPOQ rs822396* |  | AA | AG | GG | AA | AG | GG |
| Beebe-Dimmer | 2010 | 79 | 48 | 4 | 209 | 111 | 12 |
| Kaklamani | 2011 | 335 | 90 | 29 | 301 | 123 | 9 |
| *ADIPOQ rs12495941* |  | GG | GT | TT | GG | GT | TT |
| Dhillon | 2011 | 508 | 539 | 162 | 496 | 531 | 148 |
| *ADIPOQ rs168681205* |  | GG | GA | AA | GG | GA | AA |
| Dhillon | 2011 | 1077 | 161 | 5 | 1079 | 138 | 7 |
| *ADIPOQ rs168681209* |  | CC | CA | AA | CC | CA | AA |
| Dhillon | 2011 | 1014 | 206 | 13 | 986 | 230 | 11 |
| *ADIPOQ rs17366568* |  | GG | GA | AA | GG | GA | AA |
| Dhillon | 2011 | 920 | 282 | 17 | 924 | 256 | 13 |
| *ADIPOQ rs17366743* |  | TT | TC | CC | TT | TC | CC |
| Moore | 2009 | 891 | 60 | 0 | 806 | 49 | 1 |
| *ADIPOQ rs2082940* |  | CC | CT | TT | CC | CT | TT |
| Dhillon | 2011 | 969 | 226 | 14 | 907 | 260 | 26 |
| *ADIPOQ rs3774261* |  | GG | GA | AA | GG | GA | AA |
| Dhillon | 2011 | 462 | 596 | 183 | 441 | 574 | 193 |
| *ADIPOQ rs3774262* |  | GG | GA | AA | GG | GA | AA |
| Gu | 2014 | 461 | 385 | 71 | 493 | 426 | 117 |
| *ADIPOQ rs3821799* |  | CC | CT | TT | CC | CT | TT |
| Dhillon | 2011 | 383 | 601 | 249 | 350 | 606 | 259 |
| *ADIPOQ rs7639352* |  | CC | CT | TT | CC | CT | TT |
| Dhillon | 2011 | 652 | 453 | 98 | 650 | 428 | 94 |
| *ADIPOQ rs822391* |  | TT | TC | CC | TT | TC | CC |
| Dhillon | 2011 | 855 | 346 | 47 | 779 | 389 | 52 |
| *ADIPOQ rs822393* |  | CC | CT | TT | CC | CT | TT |
| Moore | 2009 | 346 | 443 | 150 | 307 | 383 | 159 |
| *ADIPOR1 rs10920531* |  | AA | AC | CC | AA | AC | CC |
| Beebe-Dimmer | 2010 | 41 | 66 | 24 | 110 | 151 | 71 |
| Kaklamani | 2011 | 89 | 204 | 158 | 72 | 207 | 148 |
| Dhillon | 2011 | 170 | 563 | 456 | 73 | 545 | 448 |
| *ADIPOR1 rs7539542* |  | GG | GC | CC | GG | GC | CC |
| Beebe-Dimmer | 2010 | 54 | 56 | 19 | 140 | 133 | 49 |
| Kaklamani | 2011 | 43 | 226 | 183 | 45 | 193 | 194 |
| Dhillon | 2011 | 538 | 513 | 135 | 543 | 489 | 135 |
| *ADIPOR1 rs12733285* |  | TT | TC | CC | TT | TC | CC |
| Kaklamani | 2011 | 48 | 221 | 183 | 71 | 222 | 145 |
| Dhillon | 2011 | 139 | 547 | 562 | 118 | 528 | 577 |
| *ADIPOR1 rs1342387* |  | CC | CT | TT | CC | CT | TT |
| Beebe-Dimmer | 2010 | 41 | 59 | 31 | 87 | 172 | 74 |
| Kaklamani | 2011 | 116 | 218 | 112 | 107 | 209 | 122 |
| *ADIPOR1 rs2232853* |  | GG | GA | AA | GG | GA | AA |
| Beebe-Dimmer | 2010 | 96 | 33 | 1 | 262 | 71 | 4 |
| Kaklamani | 2011 | 200 | 192 | 58 | 142 | 170 | 124 |
| *ADIPOR1 rs16850799* |  | CC | CT | TT | CC | CT | TT |
| Dhillon | 2011 | 751 | 392 | 65 | 713 | 405 | 67 |
| *ADIPOR2 rs1029629* |  | TT | TG | GG | TT | TG | GG |
| Dhillon | 2011 | 597 | 499 | 111 | 568 | 510 | 112 |
| *ADIPOR2 rs1044471* |  | CC | CT | TT | CC | CT | TT |
| Dhillon | 2011 | 334 | 571 | 317 | 336 | 600 | 282 |
| *ADIPOR2 rs1044825* |  | TT | TG | GG | TT | TG | GG |
| Dhillon | 2011 | 409 | 569 | 225 | 388 | 592 | 208 |
| *ADIPOR2 rs1058322* |  | CC | CT | TT | CC | CT | TT |
| Dhillon | 2011 | 598 | 497 | 104 | 568 | 484 | 120 |
| *ADIPOR2 rs10773983* |  | GG | GA | AA | GG | GA | AA |
| Dhillon | 2011 | 553 | 538 | 118 | 546 | 527 | 114 |
| *ADIPOR2 rs10773986* |  | AA | AG | GG | AA | AG | GG |
| Dhillon | 2011 | 582 | 487 | 124 | 557 | 509 | 117 |
| *ADIPOR2 rs11061946* |  | CC | CT | TT | CC | CT | TT |
| Dhillon | 2011 | 1055 | 180 | 5 | 1061 | 152 | 8 |
| *ADIPOR2 rs11061973* |  | GG | GA | AA | GG | GA | AA |
| Dhillon | 2011 | 870 | 319 | 29 | 859 | 301 | 29 |
| *ADIPOR2 rs12826079* |  | CC | CT | TT | CC | CT | TT |
| Dhillon | 2011 | 1079 | 154 | 10 | 1066 | 155 | 6 |
| *ADIPOR2 rs7967137* |  | TT | TC | CC | TT | TC | CC |
| Dhillon | 2011 | 983 | 250 | 19 | 952 | 252 | 19 |
| *ADIPOR2 rs7975600* |  | AA | AT | TT | AA | AT | TT |
| Dhillon | 2011 | 943 | 276 | 30 | 894 | 296 | 32 |

^#^ S stands for short allele, and L stands for long allele.
